# Rare coinheritance of hemoglobin vancleave with severe beta-thalassemia mutation in a patient with secondary erythrocytosis

**DOI:** 10.1038/s41439-024-00275-y

**Published:** 2024-04-23

**Authors:** Nur Aisyah Aziz, Nurul Hidayah Musa, Melina Mathews, Komalah Thevii Rajenderan, Faidatul Syazlin Abdul Hamid, Syahzuwan Hassan, Syahira Lazira Omar, Wan Nurul Afiqha Binti Wan Yusoff, Melanie Ling Binti Mohd Din, Nurul Amira Binti Jamaludin, Wan Rohani Wan Taib, Ezalia Esa, Norafiza Mohd Yasin

**Affiliations:** 1 Hematology Unit, Cancer Research Center (CaRC), Institute for Medical Research (IMR), National Institute of Health (NIH), Ministry of Health (MOH), Setia Alam, Shah Alam, Selangor Malaysia; 2 Faculty of Health Science, University Sultan Zainal Abidin, Kuala Terengganu, Terengganu Malaysia; 3 Simee Health Clinic, Ipoh, Perak Malaysia; 4 Department of Pathology, Hospital Raja Permaisuri Bainun, Ipoh, Perak Malaysia

**Keywords:** Genetic testing, DNA sequencing

## Abstract

Hemoglobin (Hb) Vancleave (NM_000518.5:c.431 A > T; dbSNP: rs33918338) is an extremely rare structural hemoglobin variant worldwide, and studies are limited. This report describes the case of a 16-year-old male patient who presented with secondary erythrocytosis. The diagnosis of Hb Vancleave, in combination with codon 41/42 (-TTCT) (NM_000518.5:c.126_129del; dbSNP: rs80356821), was confirmed by direct sequencing. This report highlights the importance of sequencing in the differential diagnosis of beta-thalassemia syndrome in Malaysia.

Hemoglobin abnormalities are caused by molecular changes involving two primary globin gene clusters (alpha and/or beta) located on chromosomes 11 (beta; 11p15.4; OMIM# 141900)^1^ and 16 (alpha; 16p13.3; OMIM# 141800 and 141850)^2^. The clinical consequences are associated with qualitative (hemoglobinopathies) and/or quantitative (thalassemia) defects in globin chain expression^3^. Coinheritance of both structural hemoglobinopathies and thalassemia in a patient results in a continuum of complex phenotypes. To date, more than 100 high-oxygen-affinity hemoglobinopathies have been described worldwide, and their presence usually leads to erythrocytosis. Although patients with erythrocytosis are generally asymptomatic, identifying patients with high oxygen affinity is important in young patients or those with high hemoglobin concentrations and a family history of polycythemia^4^. A correct diagnosis is important for the clinical management and long-term outcome of patients^4–6^.

Hemoglobin Vancleave (HGVS: NM_000518.5:c.431A > T; dbSNP: rs33918338) is an extremely rare beta-globin chain variant, and its prevalence is unknown. Two entries for Hb Vancleave were found in public databases (HbVar ID: 1234 and IthaID: 1298), but no clinical case reports were found in the literature. The molecular basis of Hb Vancleave is based on a single-nucleotide substitution at codon 143 (CAC > CTC) of the *HBB* gene, which changes the coding amino acid from histidine (His) to leucine (Leu). Theoretically, the side chain difference between leucine and histidine will affect the biochemical characteristics of Hb Vancleave relative to normal hemoglobin. In the normal hemoglobin tetramer, histidine has a positively charged imidazole side chain, which is one of the vital components of the binding site for 2,3-diphosphoglycerate (2,3-DPG), which is essential for regulating the affinity of hemoglobin for oxygen. In contrast, in Hb Vancleave, histidine is substituted with leucine, which contains an isobutyl side chain that converts the 2,3-DPG binding site to a nonpolar form. This subsequently modifies the oxygen affinity of hemoglobin. The same effects of high oxygen affinity were also observed for Hb Abruzzo^7^, Hb Syracuse^8^, and Hb Little Rock^9^, which are similarly altered at codon 143 of the *HBB* gene.

Malaysia is a tropical country in Southeast Asia where multiethnic populations and mixed marriages are common. Thus, complex genetic interactions from different ethnic backgrounds cause a wide clinical spectrum of hemoglobin disorders in Malaysia. Most of the patients registered in the Malaysian Thalassemia Registry were Malays (63.95%), Chinese (11.75%), and Kadazan-Dusuns (11.36%). However, hemoglobin disorders have rarely been reported among Malaysian Indians^10,11^. Several clinically important mutations in the *HBB* gene have been reported in major ethnic groups in Malaysia. Codon 26 (GAG > AAG) Hb E, codon 41/42 (–TTCT), IVS 1–1 (G > T), and IVS 1–5 (G > C) are the most common disease-associated *HBB* gene variations in Malays, while codon 41/42 (–TTCT) and IVS 2–654 (C > T) were predominantly detected among the Malaysian Chinese population with beta-thalassemia^12^. Here, we report the first case of Hb Vancleave combined with a severe beta-thalassemia mutation associated with secondary erythrocytosis in a Malaysian Indian teenager.

A 16-year-old student (RS873/23) of Indian Sikh descent initially volunteered to participate in the National Thalassemia Screening Program for secondary school students in Malaysia. His parents provided informed consent for the screening program at his school. Peripheral blood samples and data were collected following the Declaration of Helsinki and the Malaysian National Institute of Health (NIH) guidelines for the conduct of research at Ministry of Health (MOH) institutions and facilities.

The patient was a smoker and currently vapes. At presentation, he was asymptomatic, with a Hb level of 18.3 g/dL. No history of transfusion was reported. The peripheral blood film (PBF) showed packed red cells with hypochromic microcytic erythrocytes for his age. He has two siblings, including his younger sister, who is also asymptomatic. His parents were not consanguineous. His father is a Malaysian Sikh who died at the age of 51 from heart disease and dyslipidemia. No paternal history of thalassemia was reported. His mother is a 42-year-old Indian woman from Uttar Pradesh, India, and she has three siblings who were suspected of having thalassemia, but the diagnosis was never confirmed. In 2014, it was suspected that she had thalassemia during pregnancy, yet no confirmatory test was performed. The family tree of patient RS873/23 is shown in Fig. 1A.

**Fig. 1:**
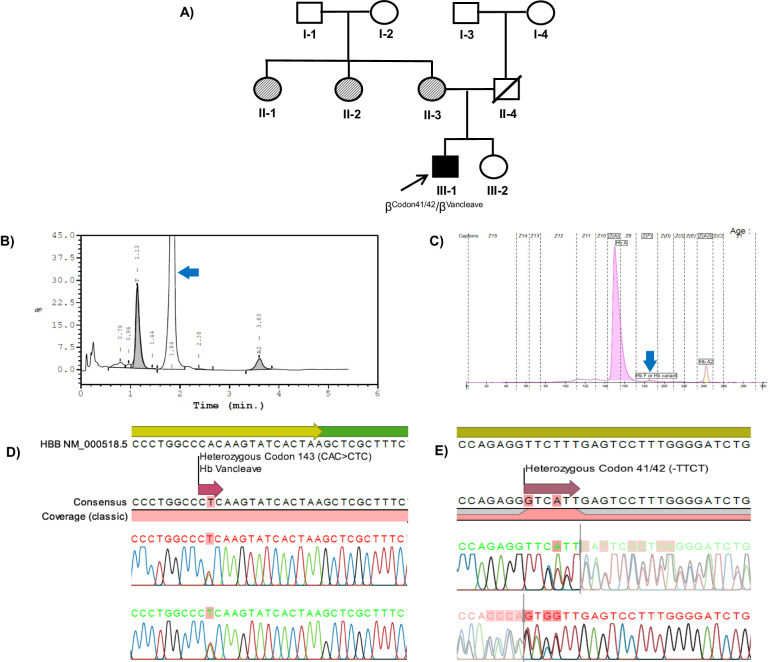
Family tree, hemoglobin analysis, and sequencing chromatograms of thecase report (III-1; RS873/23). **A** Three-generation family tree of case RS873/23 shows both affected and asymptomatic family members. The arrow symbol marked the index case (III-1; RS873/23). Squares indicate male, circles indicate female, upward diagonal stripes indicate affected/ suspected proband, black-filled symbols indicate affected proband with compound heterozygous states of β^Codon41/42^/β^Vancleave^ genotype, and white symbols indicate unaffected proband. **B** High-performance liquid chromatography shows mildly increased HbA_2_, high HbF, and unknown variants. **C** Capillary electrophoresis shows a peak for HbA, HbA_2_, and HbF/Hb-variant. The blue arrow indicates the presence of the Hb variant. **D**, **E** show sequencing analysis of the HBB gene revealed double heterozygotes for Hb Vancleave (Codon 143 [CAC>CTC]) and Codon 41/42 (-TTCT), respectively. The top bar panel with reference sequence; yellow indicates the *HBB* transcript region, green indicates the HBB gene untranslated region, the second bar panel with consensus HBB gene sequence from case III-1, and the third- and fourth-panel sequencing chromatograms from the reverse and forward primers of the HBB gene, respectively. The red arrow indicates the mutant sequence.

The peripheral blood of patient RS873/23 was collected for full blood count (FBC) and hemoglobin analysis (Hb analysis). Both FBC and Hb analyses were performed in primary health care facilities. His FBC showed hypochromic microcytic erythrocyte indices, with a red blood cell (RBC) count, mean corpuscular volume (MCV), mean corpuscular hemoglobin (MCH), and red cell width (RDW) of 8.94 × 10^6^/µL, 64.2 fL, 20.5 pg and 20.4%, respectively. The peripheral blood film (PBF) showed packed erythrocytes with a hypochromic microcytic morphology. Hemoglobin analysis by capillary electrophoresis (CE; Sebia, France) revealed elevated HbA_2_ (4.2%) and normal HbA (82.3%) levels (Fig. 1B). An abnormal HbF peak was detected by CE. High-performance liquid chromatography (HPLC; Bio-Rad, USA) revealed that 76.1% of the hemoglobin variants were abnormal, with a retention time of 1.84 min. The HbA_2_ level was borderline high (3.7%), and the HbF level was elevated (16.8%) (Fig. 1C). The results of hemoglobin analysis suggested hereditary persistence of fetal hemoglobin (HPFH) or delta-beta-thalassemia.

Molecular investigations to detect common and unknown beta-globin gene cluster deletions that are associated with HPFH and delta-beta-thalassemia phenotypes were carried out by using multiplex gap polymerase chain reaction (MGap-PCR) and multiplex ligation probe-dependent amplification (MLPA). However, no deletions were detected. Therefore, we suspected that this patient might have compound or homozygous *HBB* gene mutations that can cause both elevated HbF (16.8%) and an abnormal peak (76.1%) at a retention time of 1.84 min on HPLC. Direct sequencing was carried out to detect disease-associated variations in the beta-globin gene (*HBB*; RefSeq ID: NM_000518.5), and the results confirmed the presence of compound heterozygous variants of codon 41/42 (-TTCT) [HGVS: (NM_000518.5:c.126_129del)] (Fig. 1D) and codon 143 (CAC > CTC) Hb Vancleave (Fig. 1E). A summary of the hematology and molecular test results in this study is provided in Table 1.

**Table 1:** Hematology and DNA analysis findings for the case RS873/23.

Parameters	RS873/23
Age (year)/sex	16/Male
Hb (g/dL)	18.3
RBC (x10^6^/uL)	8.94
Hct (%)	38.1
MCV (fL)	64.2
MCH (pg)	20.5
MCHC (g/dL)	31.9
RDW-CV (%)	20.4
**Capillary electrophoresis**	
HbA (%)	82.3
HbA2(%)	4.2
HbF (%)	–
HbE (%)	–
**High-performance liquid chromatography**	
HbA (%)	0.4
HbA2/E (%)	3.7
HbF (%)	16.8
Hb Others (%)	76.1
**DNA analysis**	
β-MGap	β/β
β-MLPA	β/β
β-sequencing	β^Codon41/42^/β^Vancleave^
α-MLPA	αα/αα
α-sequencing	αα/αα

β^Codon41/42^: Deletion variant at codons 41/42 (-TTCT) (NM_000518.5:c.126_129del) of *HBB* gene, β^Vancleave^: Single nucleotide substitution at codon 143 (CAC > CTC) (NM_000518.5:c.431 A > T) of *HBB* gene; Hb Vancleave, αα/αα: normal copy of *HBA1/2* genes, β/β: normal copy of *HBB* gene.

Our case is an incidental finding from the National Thalassemia Screening Program in Malaysia. This is the first case report in the literature and the first identification of Hb Vancleave in the Malaysian population. The majority of hemoglobin disorders are inherited as autosomal recessive. However, in some cases, disease-associated genetic variations in globin gene clusters can be caused by de novo or dominant genetic variations. In this study, we identified a case of a Malaysian patient with compound heterozygous variants of Hb Vancleave and codon 41/42 (-TTCT), which are rare among Indian Sikh in Malaysia. The inheritance pattern of these variations cannot be determined because parental samples were not available. Nevertheless, we speculate that his father might be homozygous for Hb Vancleave, which could cause polycythemia and hyperviscosity syndrome; this condition might have led to his heart problems and subsequent death at the age of 51 years, which is considered young for heart disease and complications of cardiomyopathy. The clinical phenotypes were comparable to those of another patient with high oxygen affinity—a 4-year-old Malay girl with homozygous Hb Tak who presented with symptomatic polycythemia and congestive heart failure^13^. Hb Tak is mildly unstable; thus, patients in a homozygous state are more symptomatic and present with symptoms early in life.

A common disease-associated frameshift variation at codon 41/42 (-TTCT) compounded with Hb Vancleave was detected in our index patient. The four-nucleotide deletion between codons 41 and 42 of the *HBB* gene results in a stop codon at codon 59, which subsequently terminates translation and reduces the rate of beta-globin chain synthesis. The genetic alteration of codon 41/42 (-TTCT) has been reported in many populations, including Chinese, Malaysian, Singaporean, Taiwanese, Thai, Punjabi, Pakistani, Japanese, Korean, English, and Indonesian populations^14^. Particularly in Malaysia, the codon 41/42 (-TTCT) variation is commonly found in Malaysian Chinese and Malays but has never been reported in Malaysian Indians^12^. We noted that the patient’s mother was originally from Uttar Pradesh, India, which has a high prevalence of beta-thalassemia with codon 41/42 (-TTCT) being one of the most common beta-thalassemia alleles^15^. Hence, based on the family history of the index patient, we postulate that codon 41/42 (-TTCT) and Hb Vancleave could have been inherited from the maternal and paternal sides, respectively. In addition, although both the Hb Vancleave and codon 41/42 (-TTCT) alleles were functionally altered, the Hb value was still high, likely due to physiological compensation from smoking and vaping habits.

Differential diagnosis of high oxygen affinity may be laborious and complex due to its plethora of clinical presentations and its unknown prevalence in the population^5,16^. Particularly in Malaysia, where hemoglobin disorders are highly prevalent, patients with erythrocytosis should be prioritized for hemoglobin anomaly investigations via direct DNA sequencing. This is an important step to ensure that clinical management is successful and that effective investigations can be performed for patients with erythrocytosis^5,17^. Previous studies have also shown that coinheritance of high-affinity hemoglobin with delta-beta thalassemia or beta-thalassemia leads to marked polycythemia; otherwise, patients are clinically healthy, but the risk of thrombosis increases later in life^4^. In addition, Percy and colleagues recommended that if the P50 value is not available, HPLC and Sanger sequencing can be used as alternative methods for the differential diagnosis of high-affinity hemoglobin. Erythrocytosis with concomitant iron deficiency can normalize hemoglobin levels and therefore make it difficult to detect high-affinity hemoglobin by performing only routine laboratory tests^18^. Some of the silent variants may be overlooked in the presumptive diagnosis. Hence, when encountering these atypical cases, molecular DNA analysis is recommended^17,19^. In summary, this is the first reported case of Hb Vancleave, a rare variant worldwide, in Malaysia. The present study highlights the importance of hemoglobin disorder awareness and the advantage of DNA analysis for patients with erythrocytosis, which can be challenging due to the clinical and genetic heterogeneity of thalassemia syndrome in Malaysia.

## HGV Database

The relevant data from this Data Report are hosted at the Human Genome Variation Database at 10.6084/m9.figshare.hgv.3391. 10.6084/m9.figshare.hgv.3394.
